# A systematic discrepancy between the short circuit current and the integrated quantum efficiency in perovskite solar cells

**DOI:** 10.1038/s41467-023-41263-0

**Published:** 2023-09-06

**Authors:** Michael Saliba, Eva Unger, Lioz Etgar, Jingshan Luo, T. Jesper Jacobsson

**Affiliations:** 1 IEK5-Photovoltaics, Forschungszentrum Jülich, Jülich, Germany; 2 Institute for Photovoltaics (IPV), University of Stuttgart, Stuttgart, Germany; 3 Young Investigator Group Hybrid Materials Formation and Scaling, Helmholtz-Zentrum Berlin für Materialen und Energie GmbH, Berlin, Germany; 4 Chemical Physics and NanoLund, Lund University, Lund, Sweden; 5 Institute of Chemistry, the Center for Nanoscience and Nanotechnology, The Hebrew University of Jerusalem, Jerusalem, Israel; 6 Institute of Photoelectronic Thin Film Devices and Technology, Key Laboratory of Photoelectronic Thin Film Devices and Technology of Tianjin, College of Electronic Information and Optical Engineering, Nankai University, Tianjin, 300350 China

**Keywords:** Materials for energy and catalysis, Energy science and technology

## Abstract

Halide perovskites solar cells are now approaching commercialisation. In this transition from academic research towards industrialisation, standardized testing protocols and reliable dissemination of performance metrics are crucial. In this study, we analyze data from over 16,000 publications in the Perovskite Database to investigate the assumed equality between the integrated external quantum efficiency and the short circuit current from JV measurements. We find a systematic discrepancy with the JV-values being on average 4% larger. This discrepancy persists across time, perovskite composition, and device architecture, indicating the need to explore new perovskite physics and update reporting protocols and assumptions in the field.

## Abstract (web summary)

Defining and revising best practices for accurate measurements and data reliability and quality checks is a continuous effort for the commercialization of perovskite solar cells. Here, the authors discuss the systematic discrepancy between the short circuit current and integrated quantum efficiency.

## Introduction

In the last decade, halide perovskites have emerged as a class of promising solar cell materials. During this time, record efficiencies have surpassed 25%^1,2^ and the research has gone from basic research to gradually also containing more technology-oriented device development. Several companies now claim that commercial perovskites solar cells may be just around the corner^3–5^. Those early-stage commercialization efforts increase the demand for rigorous performance evaluation and standardized testing protocols. Accurate measurements and dissemination of reliable performance metrics are in fact vital for providing a truthful reflection of the field’s progress and necessary for guiding the development of commercial products. In this regard, the publication of erroneous and overinflated performance metrics is damaging the exploitation of any technology as it causes misled research efforts, diminishes the trustworthiness of the research field, and hampers the development of commercial devices.

For emerging PV materials, where device efficiency is a key performance indicator and the incentive to demonstrate high numbers is strong, history has shown us reasons to be concerned^6–8^. There is, for example, data indicating that the performance of organic solar cells systematically has been overestimated in the literature^6^. Probably less due to fraudulent intent and more due to a lack of unified testing protocols and appropriate check-ups. There are in fact several pitfalls when evaluating solar cell performance^9,10^, e.g. lamp calibration, spectral mismatch, light inhomogeneities, leakage currents due to improper masking, etc. Maybe are there also too little incentives to go the extra mile to ensure correctly calibrated measurements, especially if it would result in slightly lower values which may impede publication. Early development and work not explicitly focusing on efficiency optimisation are expected to be especially susceptible to errors of this kind. There are no reasons to believe that the perovskite field would be spared from such problems. One could in fact argue that this is a good example of a field where this type of problem can be expected.

Even if not taking into account common pitfalls like the calibration of the solar simulator, proper cell masking, and the correct determination of the active cell area^9^, the perovskites have their own set of peculiarities. Perovskites are for example known to show hysteresis in the JV response^11–13^, an initial burn-in^14^, and for having stability problems^15–17^. All of this can distort the performance metrics obtained from JV scans.

Those problems have not gone unnoticed. The research community has responded appropriately and there is in fact an entire sub-genre dedicated to discussing, evaluating, and proposing measuring protocols for emerging PV materials, both for general solar cell evaluation^9,10,18^, and for perovskites specifically^19–23^. Publishers are also more frequently pushing for the dissemination of detailed measurement protocols, as well as for the underlying raw data. Both the Nature journals and Energy and Environmental Science have for example implemented checklists to be used for anyone wanting to publish perovskite device data^24–26^, to mention just two prominent examples.

Measurement protocols and their underlying assumptions are, however, not static but gradually evolve as new pitfalls and peculiarities of the investigated systems are discovered. In this paper, we take a closer look at one of those quality checks, namely the integrated external quantum efficiency, *J*_*sc,EQE*_, and how it compares to the short circuit current extracted from JV-measurements, *J*_*sc,JV*_.

The standard way to evaluate solar cell performance is by a JV measurement. Due to known problems, like calibration uncertainties, spectral mismatch, and hysteresis, a comparison with a second type of independent measurement highly improves the trustworthiness of the results. Certification at an independent test institute is considered the gold standard. That is, however, not attainable for routine experiments. To verify that the determined PCE from a JV measurement reflects the steady-state performance of the solar cell, reference measurements carried out under steady-state conditions are therefore recommended. Two such options are external quantum efficiency, EQE, commonly carried out at a steady bias, i.e., 0 V, and stabilised efficiency during maximum power point tracking (MPPT), where the latter aims to mimic operational conditions.

## Observed mismatch between integrated EQE and J_SC_

If the EQE is measured as a function of wavelength, the response can be multiplied with the *AM 1.5* spectrum and the unit charge, *q*, and integrated over the entire spectra. This gives an estimate of the short circuit current under illumination, *J*_*sc,EQE*_, (Eq. 1), which can be compared to the short circuit current extracted from the JV-measurement, *J*_*sc,JV*_.

1 $${J}_{{sc},{EQE}}={\int }_{0}^{{{\infty }}}{qEQE}\left(\lambda \right)S\left(\lambda \right)d\lambda,\, S={AM}1.5$$

The underlying assumption is that *J*_*sc,EQE*_ and *J*_*sc,JV*_ should be the same, or at least quite similar and that a large mismatch indicates that something is not right^19^. This check is a part of the checklist in Energy and Environmental Science^25^, and the reporting standards of the Emerging-PV Reports Initiative^27^. Meanwhile, when submitting to Nature journals, the community is required to provide details of characterization, or experimental and analytical design in the solar cells reporting summary, where they are being asked if a comparison of the two values has been made^26^. The equivalence of *J*_*sc,EQE*_ and *J*_*sc,JV*_ is considered as a robust metrics, at least for established PV technologies. It is for example the basis of one of the most common procedures of calibrating solar simulators, where its light intensity is tuned until the *J*_*sc,JV*_ for a reference silicon cell matches its *J*_*sc,EQE*_^9^. A *J*_*sc,EQE*_ and *J*_*sc,JV*_ discrepancies have also been used to detect questionable data in the organic PV-community^6^.

For perovskite cells, concerns have been raised that this may be a less reliable test than assumed. In 2020, Saliba et al. pointed out that for externally certified cells reported in the literature, *J*_*sc,JV*_ appears to be systematically larger than *J*_*sc,EQE*_^28^. The dataset was, however, small and the underlying papers do not always clearly state if the EQE was measured on the exact same devices as the corresponding certified JV data. This was an indication of a mismatch between the two metrics, but with insufficient data to turn the intuition into a solid conclusion.

Here we can now demonstrate that for perovskite cells there really is a statistically significant discrepancy between *J*_*sc,EQE*_ and *J*_*sc,JV*_. This has been possible due to the Perovskite Database Project^29,30^, which is the result of a communal effort to collect all perovskite solar cell device data available in the peer-reviewed literature and make it comply with the FAIR data principles, i.e. Findable, Accessible, Interoperable, and Reusable^31,32^. In the Perovskite Database, there are at the time of writing data for over 42,000 devices, which represents essentially every device someone has thought it worth the trouble to properly describe in the peer-reviewed literature up until spring 2020.

**Fig. 1:**
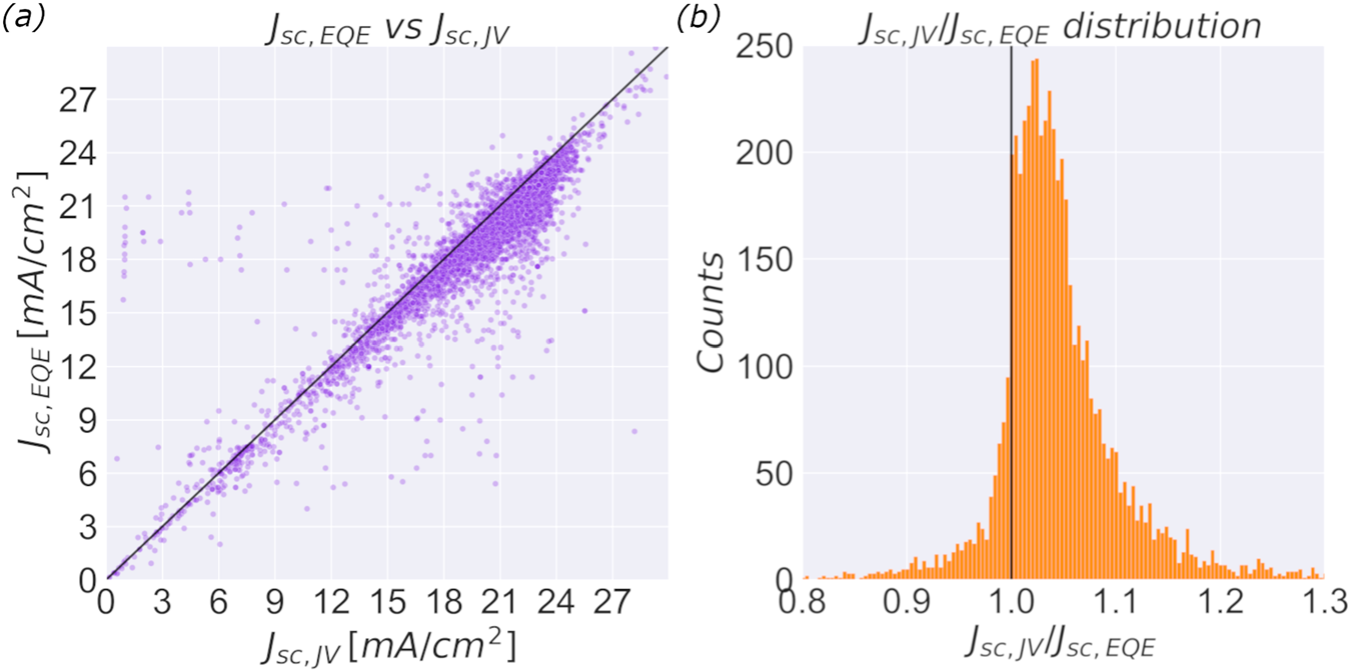
Relation between *J*_*sc,JV*_ and *J*_*sc,EQE*_. **a** Integrated external quantum efficiency, *J*_*sc,EQE*_, against short circuit current from JV-measurements, *J*_*sc,JV*_, for all 5575 devices found in the Perovskite Database where both values are reported. The black diagonal line represents *J*_*sc,JV*_ = *J*_*sc,EQE*_. **b** Distribution of *J*_*sc,JV*_/*J*_*sc,EQE*_ for the entire dataset. The bin size is set to 0.004.

Out of the >42,000 devices in the dataset, there are 5575 for which both *J*_*sc,EQE*_ and *J*_*sc,JV*_ are reported. In Fig. 1, the *J*_*sc,EQE*_ is plotted against the *J*_*sc,JV*_ for the entire dataset, including devices of all levels of performance, with all possible architectures, stack sequences, and perovskite compositions. This representation of the data shows that the two values are indeed rarely the same. *J*_*sc,JV*_ is larger than the *J*_*sc,EQE*_ for 83% of all data points.

## Possible causes for the observed mismatch

Potential reasons for the discrepancy between *J*_*sc,JV*_ and *J*_*sc,EQE*_ are manifold. First of all, measuring absolute EQE values is non-trivial. The calibration can fail. The implementation of the integration algorithm can affect the results if the distance between measurement points is too large^33^. A difference between the sun simulator and the reference AM 1.5 spectrum also has an effect, especially if the calibration diode’s absorption range is different from that of the test cell, which is the case if a silicon diode is used. This could be a problem when standard xenon lamps are used for IV measurements as they may have intensity spikes which fall well within the absorption range of silicon but are located outside the region of the absorption onset of typical perovskite compositions. If a lock-in amplifier is used, the frequency of the light modulation can affect the results as well^33–35^. Some of the observed discrepancy will certainly be due to errors like these. Even if there were numerous ways to mess up both JV and EQE measurements, we would expect such errors to be random and go in both directions. On average, we posit that random errors will average out when analyzing large datasets like this one.

To elucidate the exact physical mechanism, or mechanisms, behind this observation will require detailed experimental work beyond the scope of this report. We can, however, quantify the discrepancy. If *J*_*sc,JV*_/*J*_*sc,EQE*_ is plotted as a box diagram (Fig. 1b), a normal distribution is assumed, and obvious outlier are excluded, i.e., only include *J*_*sc,JV*_/*J*_*sc,EQE*_ = 1 ± 0.25, the distribution has a mean, *m*, of 1.041 with a standard deviation, *σ*, of 0.057 (including outliers give *m* = 1.050 and *σ* = 0.15). This translates to a 76% probability of measuring *J*_*sc,JV*_/*J*_*sc,EQE*_ above 1. Data does not perfectly follow a normal distribution but is skewed towards higher values, reflected in that over 83% of all reported data points are above 1. The historical data thus indicate that for a random device one would expect *J*_*sc,JV*_ to be around 4% larger than *J*_*sc,EQE*_ if both device and measurements are sound. A few measurements of *J*_*sc,JV*_/*J*_*sc,EQE*_ around 1.04 would not be a sign of a systematic discrepancy, especially given that it is not uncommon to explain away a 5% mismatch as an acceptable calibration error. When all data are considered together, the mismatch is, however, obvious.

Several possible hypotheses can be discarded based on the dataset. The cell efficiency, *PCE*, does for example have no effect on the average *J*_*sc,JV*_/*J*_*sc,EQE*_. For the worst cells, i.e. *PCE* < 5%, the data scatters widely, but for better cells the only change seen is a decrease in the spread of the *J*_*sc,JV*_/*J*_*sc,EQE*_ values with increased *PCE* (Supplementary Fig. 1). There is no systematic shift of the *J*_*sc,JV*_/*J*_*sc,EQE*_ values with respect to the open circuit voltage, *V*_*oc*_, or the fill factor, *FF*, either (Supplementary Fig. 2–3). When the short circuit current *J*_*sc,JV*_ changes, the same average value around 1.04 is seen except for the lowest and the very highest currents. For low *J*_*sc,JV*_, the data is scarce and a lot of things can go wrong in a failed device. For the highest reported *J*_*sc,JV*,_ the data is also scarce and those are reasonably the values with the highest probability of being erroneously high, as seen by the fact that some of those values are approaching, or even exceeding, the Shockley–Queisser limit.

The perovskite cells are known for that there can be hysteresis during JV-sweeps. That could obstruct the accurate determination of cell performance^11–13^, and at first glance that could be a reasonable hypothesis for the *J*_*sc,JV*,_ *J*_*sc,EQE*_ mismatch. The amount of hysteresis in the JV-measurement does, however, not affect the average *J*_*sc,JV*_/*J*_*sc,EQE*_ values (Supplementary Fig. 5). At least not to the extent the hysteresis is reflected in the JV-measurement, which may not always be the case as the JV-sweep parameters could be selected to minimize the measured hysteresis without reflecting any true steady-state conditions. The carrier extraction at the charge selective contacts does not have an effect either. The same average values for *J*_*sc,JV*_/*J*_*sc,EQE*_ are observed for all common hole and electron transport layers (Supplementary Fig. 8–9). That is also true for both nip and pin device architectures (Supplementary Fig. 10). Only for stack layers with few reported cells do we observe a larger spread in values. That is statistically expected, but even there the average *J*_*sc,JV*_/*J*_*sc,EQE*_ values are positive.

**Fig. 2:**
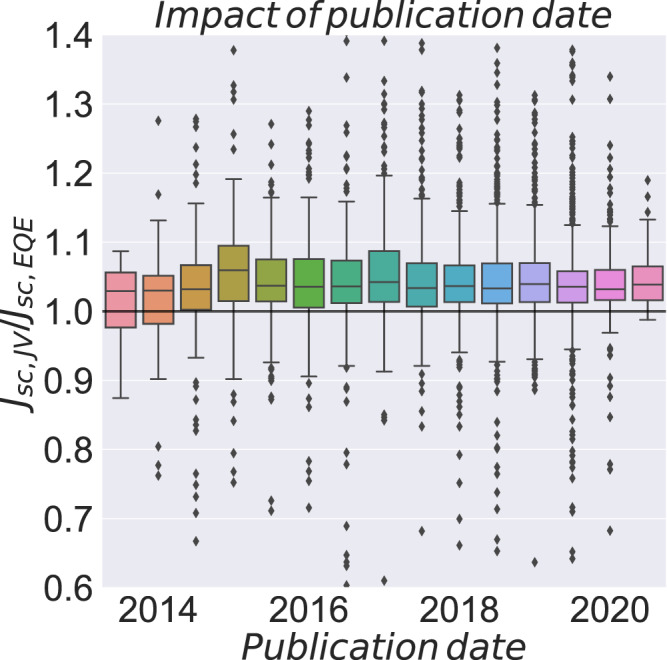
Development of the *J*_*sc,JV*_/*J*_*sc,EQE*_ as a function of publication date. The bin size is half a year. The end of the boxes represents the 25 and the 75 percentiles. The whiskers are placed at an interquartile range of 1.5, which means that for a normal distributed data set, 99.3% of points should be within that range.

If the current mismatch would be a result of measurement or calibration errors, one would expect those to decrease with time as groups improve on their artesian handicraft and their experimental protocols, as knowledge spreads through the community, and as more rigorous measurement protocols are followed. To our surprise, this is not the case (Fig. 2).

Given the perovskites stability problems, some test cells may have degraded between measurements which would give a lower *J*_*sc,EQE*_ as that measurement most often is done after the JV-measurement. The time consistency of the effect does, however, talk against that as a main exploration given the gradual increase in stability seen due to for example perovskite composition engineering.

The observed short circuit current mismatch is thus probably not due to measurement errors. It does not depend on device performance, the cell architecture, or the choice of charge-selective contacts. It has also been persistent over almost a decade of development. This makes it reasonable to assume that this effect either has something to do with the perovskite itself or that there is something in the measurement situation that makes the perovskite behave differently in typical JV and EQE measurements.

In terms of the perovskite, the composition and the band gap does not seem to have an impact. This is at least true for all compositions and band gap ranges where there is sufficient data for reliable statistics (Supplementary Fig. 7). For the highest band gap devices, i.e., above 2.4 eV, the effect seems to disappear, but in that band gap range there are too few data points to draw any firm conclusions. The largest spread in the data we have found is with respect to the perovskite deposition method, but a larger spread in the *J*_*sc,JV*_/*J*_*sc,EQE*_ values is only seen in cases where the data points are few (Supplementary Fig. 12).

In terms of the measurement conditions, in a JV-sweep, the voltage is constantly changed while it is held constant during each EQE measurement. The former is hence prone to be affected by capacitive or “poling” effects caused by the voltage sweep, which get mobile ions to move around in the applied electric field causing both capacitive effects and charge accumulation at interfaces^36,37^. A JV measurement is also generally much faster than an EQE measurement, at least historically. Another set of possible explanations revolves around that EQE often is measured without a biased light, or with a biased light much less than one sun. The photophysics of the perovskite may thus be different in the two measurements. Dye Sensitized Solar does for example tend to perform best at lower light intensities due to the photocurrent being nonlinear with respect to light intensity^38^. That could, however, not be the explanation here as that would cause the *J*_*sc,EQE*_ to be larger than *J*_*sc,JV*_^6^.

There are several things that can change in the perovskites under increased illumination. There are for example reports of light-induced phase separation^39^, light-dependent ion migration^40,41^, a large light intensity dependence of the dielectric constant^42^, as well as a strong correlation between light intensity and conductivity with up to 3-4 orders of magnitude difference between AM 1.5 and the dark^43,44^, which could cause larger resistive losses at low light conditions. The recombination pathways can also be different. The photoluminescence quantum yield has for example been found to be strongly dependent on excitation intensity, which is commonly interpreted as differences in the fraction of charge carriers recombining non-radiatively^45^, i.e., certain defect levels that cause non-radiative recombination or lead to lower extraction efficiencies may simply get saturated at higher light intensities. This points towards several plausible mechanisms that potentially could explain lower short circuit currents from low light intensity EQE measurements. The Perovskite Database does, unfortunately, not resolve the bias light conditions during EQE measurements, but based on the reasoning above, some of the most reasonable hypotheses seem to be related to light intensity-induced changes in the perovskite. Those could for example be related to differences in photo current generation, conduction, ion transport, or recombination. Resolving this will require further detailed studies.

## Alternative quality checks

An alternative quality check of the device performance is to measure stabilised efficiency, *PCE*_*stab*_, ideally under maximum power point tracking. That is a steady state measurement under operational conditions and considered to be a better measure of the true efficiency of a cell than the *PCE* extracted from a dynamic JV-scan, *PCE*_*JV*_. Not surprisingly, the *PCE*_*stab*_ is lower than *PCE*_*JV*_. For the 3367 data points where both measures are available the median difference is 2.4 % (Supplementary Fig. 13), which is in the same direction but smaller than the average discrepancy between the *J*_*sc,JV*_ and the *J*_*sc,EQE*._ If the two discrepancies, i.e. *PCE*_*JV*_/*PCE*_*stab*_ and *J*_*sc,JV*_/*J*_*sc,EQE*_ are compared, the median value of their quotient is 1.016 (Supplementary Fig. 14) indicating a co-variation.

## Summary

To summarize. Based on the historical device data in the Perovskite Database we have demonstrated a systematic discrepancy between the short circuit current extracted from JV-measurements, *J*_*sc,JV*_, and that obtained from integrating the external quantum efficiency *J*_*sc,EQE*_. The *J*_*sc,JV*_ could on average be expected to be 4% larger than the *J*_*sc,EQE*_. Based on the data in the Perovskite Database we show that this mismatch transcends device performance, cell architecture, the choice of charge selective contacts, perovskite composition and deposition procedure and that it has been persistent for almost a decade of experimental development. The exact mechanism behind this discrepancy is still unclear and requires further experimental studies.

## Conclusion

While we here could demonstrate that the short circuit current extracted from JV measurements, *J*_*sc,JV*_ on average is 4% larger than the integrated external quantum efficiency, an approximate resemblance of the two measurements is still a reasonably good consistency check. However, if the two values systematically and on average are equal, it is a high probability that at least one of the measurements is inaccurate. In other words, a higher *J*_*sc,JV*_ compared to *J*_*SC,EQE*_ is the norm rather than the exception.

One of the goals of this work is to highlight potential problems of systematic overrepresentation of performance data published on perovskite PV devices. From this metadata study, we conclude, among others, that more attention should be paid to MPP tracking as the primary performance measurement, which compared to JV-scans and EQE measurements is the measurement most closely representing relevant operational conditions.

This work also demonstrates the power of common data dissemination platforms like the Perovskite Database Project^46^, which enabled us to demonstrate new perovskite physics to explore that previously had been overlooked due to the lack of large datasets. Finally, it demonstrates that some of the perovskite communities' reporting protocols and their underlying assumptions need a slight update.

### Supplementary information

Supplementary Information

## Data Availability

The data is acquired from the Perovskite Database Project which is described in Nature Energy (10.1038/s41560-021-00941-3). The most up-to-date data set can be downloaded from https://www.perovskitedatabase.com. The specific dataset used in the analysis (downloaded from the Perovskite database in December 2021) is also archived in Zenodo (10.5281/zenodo.5837035, https://zenodo.org/record/5837035#.YfIIFv5ByUk) as well as in the Github repository linked to this paper (https://github.com/Jesperkemist/perovskitedatabase_Jsc_Jeqe_discrepancy.git).

## Code availability

The code for analysing the data and for plotting all figures are found in the Github repository, https://github.com/Jesperkemist/perovskitedatabase_Jsc_Jeqe_discrepancy.git linked to this paper. The repository also contains the data for doing the analysis. The code is also deposited at Zenodo, 10.5281/zenodo.8116724.
