# Supplementary material for: A systematic discrepancy between the short circuit current and the integrated quantum efficiency in perovskite solar cells
Source: Nat Commun. 2023 Sep 6;14:5445. doi: 10.1038/s41467-023-41263-0 (PMC10482911; doi:10.1038/s41467-023-41263-0)
Supplement: Supplementary file 1 — Supplementary Information [file 41467_2023_41263_MOESM1_ESM.pdf]

Supporting information

A Systematic Discrepancy Between the Short Circuit Current and the Integrated Quantum Efficiency in Perovskite Solar Cells

Michael Saliba*[1, 2], Eva Unger[3, 4], Lioz Etgar[5], Jingshan Luo[6], T. Jesper Jacobsson[6]

1. IEK5-Photovoltaics, Forschungszentrum Jülich, Jülich, Germany.
2. Institute for Photovoltaics (IPV), University of Stuttgart, Stuttgart, Germany.
3. Young Investigator Group Hybrid Materials Formation and Scaling, Helmholtz-Zentrum Berlin für Materialen und Energie GmbH, Berlin, Germany.
4. Chemical Physics and NanoLund, Lund University, Lund, Sweden
5. Institute of Chemistry, The Hebrew University of Jerusalem, Jerusalem, Israel
6. Institute of Photoelectronic Thin Film Devices and Technology, Key Laboratory of Photoelectronic Thin Film Devices and Technology of Tianjin, College of Electronic Information and Optical Engineering, Nankai University, Tianjin 300350, China

*Corresponding authors
M. Saliba. michael.saliba@ipv.uni-stuttgart.de
T.J. Jacobsson. jacobsson.jesper.work@gmail.com

Supplementary Figures

To explore the observed mismatch between the short circuit current extracted from JV measurements, $J_{sc,JV}$, and the integrated external quantum efficiency, $J_{sc,EQE}$, the quotient of the two currents, $J_{sc,JV}/J_{sc,EQE}$, have been compared with several different parameters in the following figures. Data is plotted both as scatterplots and as box diagrams. In all the box diagrams, the end of the boxes represents the 25 and the 75 percentiles. The whiskers outside the boxes are placed at an interquartile range of 1.5, which means that for a normal distributed data set, 99.3 % of all points should be within that range.

In fig. S.1. $J_{sc,JV}/J_{sc,EQE}$ is compared to cell efficiency. For the worst cells, i.e. PCE < 5 %, the data scatters widely, but for better cells the only observed change is a decrease in the spread of the $J_{sc,JV}/J_{sc,EQE}$ values with increased PCE.

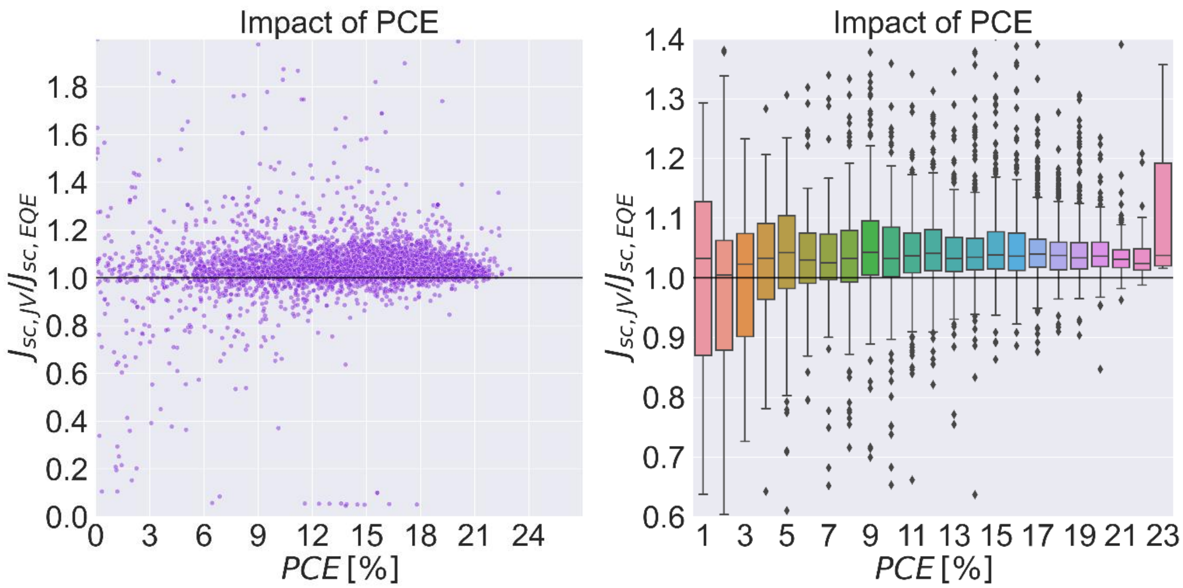

Figure S.1. Impact of *PCE*. (Left) $J_{sc,JV}/J_{sc,EQE}$ against PCE for all cells in the Perovskite Database where data for all three parameters are available. (Right). Boxplot of $J_{sc,JV}/J_{sc,EQE}$ with respect to cell efficiency. The bin size is 0.5 %.

In fig. S.2, $J_{sc,JV}/J_{sc,EQE}$ is compared to the open circuit voltage, V_{oc}, For low values, i.e. below 0.5 V, there is a large spread in the data. That is probably a result of few data points and the multitude of things that can be wrong for such poorly performing devices. For higher V_{oc} the average $J_{sc,JV}/J_{sc,EQE}$ is fairly stable around 1.04.

In fig S.3., the $J_{sc,JV}/J_{sc,EQE}$ is compared to the fill factor. By excluding lowest values of the *FF* where data is scarce and cells are hardly working, and the highest numbers, which due to physical constraint can be assumed to be erroneous data points, there is not much of a trend with respect to the average $J_{sc,JV}/J_{sc,EQE}$ values. The spread of the $J_{sc,JV}/J_{sc,EQE}$ does, however, decrease somewhat with increased FF.

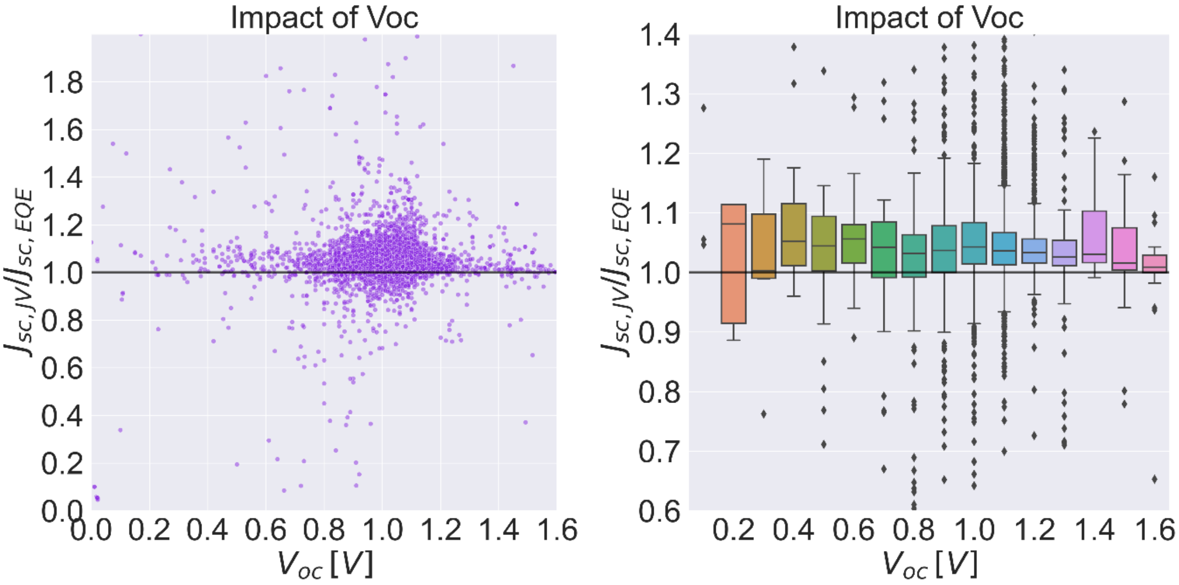

Figure S.2. Impact of V_{oc}. (Left) $J_{sc,JV}/J_{sc,EQE}$ against open circuit voltage, V_{oc}, for all cells in the Perovskite Database where data for all three parameters are available. (Right) Boxplot of $J_{sc,JV}/J_{sc,EQE}$ with respect to V_{oc}. The bin size is 0.1 V.

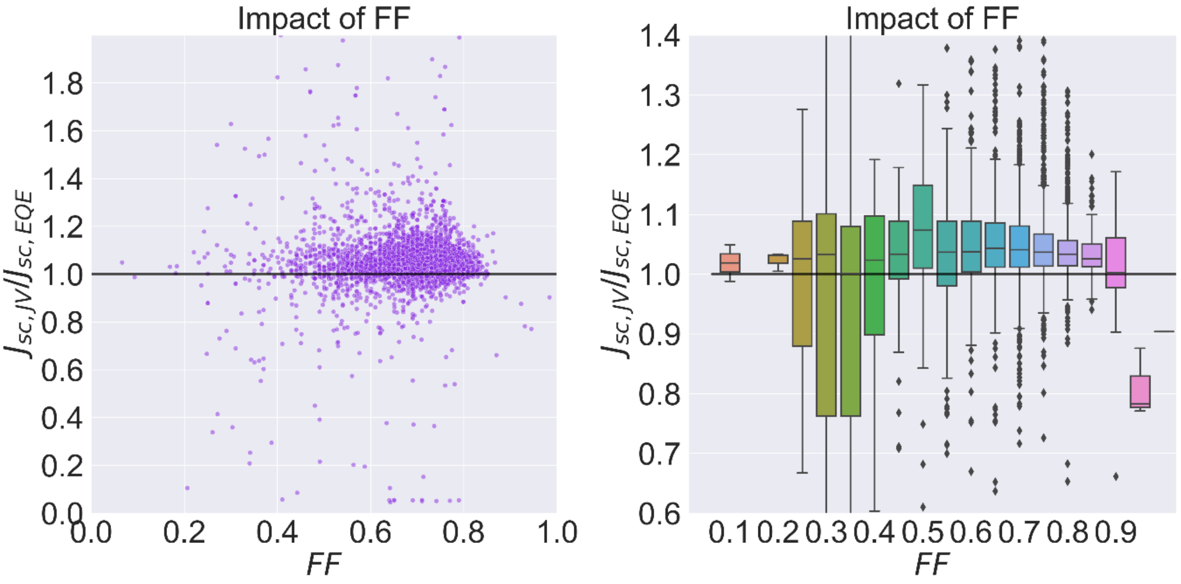

Figure S.3. Impact of *FF*. (Left) $J_{sc,JV}/J_{sc,EQE}$ against the fill factor, *FF*, for all cells in the Perovskite Database where data for all three parameters are available. (Right) Boxplot of $J_{sc,JV}/J_{sc,EQE}$ with respect to the *FF*. The bin size is 0.05.

In fig. S.4., the $J_{sc,JV}/J_{sc,EQE}$ is plotted against $J_{sc,JV}$. The same average $J_{sc,JV}/J_{sc,EQE}$ values around 1.04 are seen except for the lowest and the very highest values of $J_{sc,JV}$. For low $J_{sc,JV}$, data is scarce and devices are hardly working. For the highest reported $J_{sc,JV}$, data is also scarce and those values are reasonable ones with the highest probability of being erroneously high.

In fig. S.5., the $J_{sc,JV}/J_{sc,EQE}$ is compared to the hysteresis index, H. There are several ways to quantify the hysteresis in JV-measurements. Here we have used the definition from the Perovskite Database Project[28] which defines H, according to eq. S.1 where r refers to the reversed scan direction (i.e. V_{oc} to 0), and f refers to the forward scan direction (i.e. 0 to V_{oc}). We see the same general behaviour with average $J_{sc,JV}/J_{sc,EQE}$ values around 1.04 independent of H. That is except for strongly hysteric cells ($H > 0.5$) where the scatter in data is larger and the statistics less certain.

$$H = r_1 \cdot r_2 \cdot r_3 \cdot r_4 - 1, r_1 = \frac{Jsc_f}{Jsc_r}, r_2 = \frac{Voc_f}{Voc_r}, r_3 = \frac{FF_f}{FF_r}, r_4 = \frac{PCE_f}{PCE_r}, f_x = \frac{1}{r_x} \text{ if } r_x < 1 \quad \text{(S.1)}$$

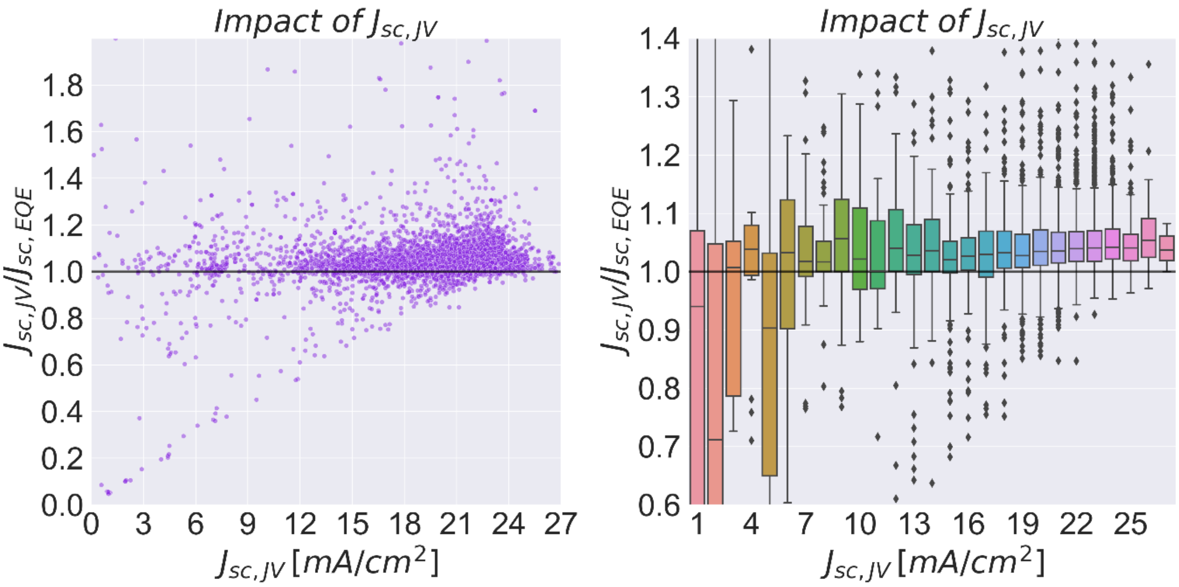

Figure S.4. Impact of J_{sc} (Left) $J_{sc,JV}/J_{sc,EQE}$ against $J_{sc,JV}$ for all cells in the Perovskite Database where data for all three parameters are available. (Right). Boxplot of $J_{sc,JV}/J_{sc,EQE}$ with respect to $J_{sc,JV}$. The bin size is 1 mA/cm^2.

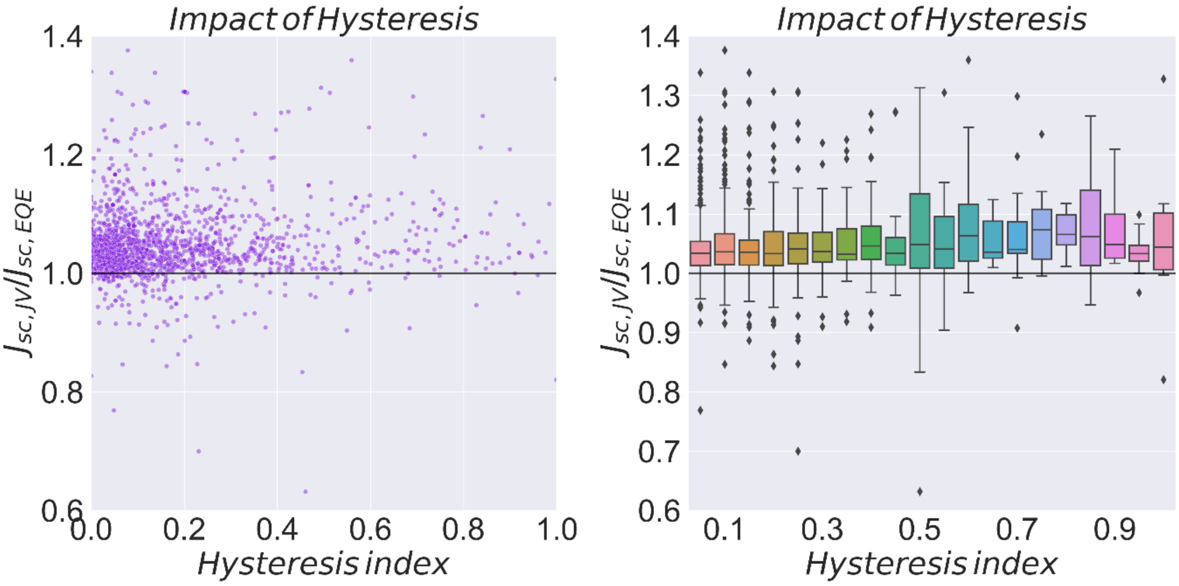

Figure S.5. Impact of Hysteresis. (Left) $J_{sc,JV}/J_{sc,EQE}$ against the hysteresis index, H, defined in the text. (Right). Boxplot of $J_{sc,JV}/J_{sc,EQE}$ with respect to H. The bin size is 0.05.

In fig. S.6, $J_{sc,JV}/J_{sc,EQE}$ is compared to the publication date. If the current mismatch would be a result of measurement or calibration errors, one would expect those to decrease with time as groups improve on their artesian handicraft and their experimental protocols, as knowledge spreads through the community, and as more rigorous measurement protocols are followed. The data in the figure demonstrate that this effect has been stable for almost a decade.

In fig S.7. $J_{sc,JV}/J_{sc,EQE}$ is plotted as a function of perovskite band gap. The band gap does not seem to have much of an impact either, at least for band gap ranges where there is sufficient data for reliable statistics.

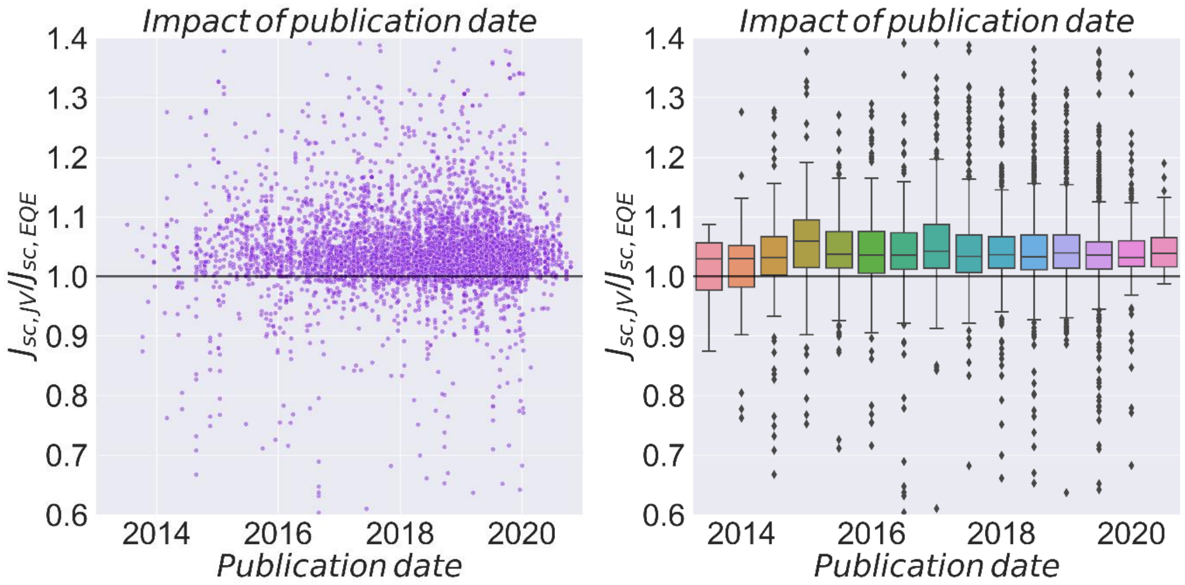

Figure S.6. Impact of publication date. (Left) $J_{sc,JV}/J_{sc,EQE}$ against the publication date. (Right) Boxplot of $J_{sc,JV}/J_{sc,EQE}$ with respect to the publication date. The bin size is six months.

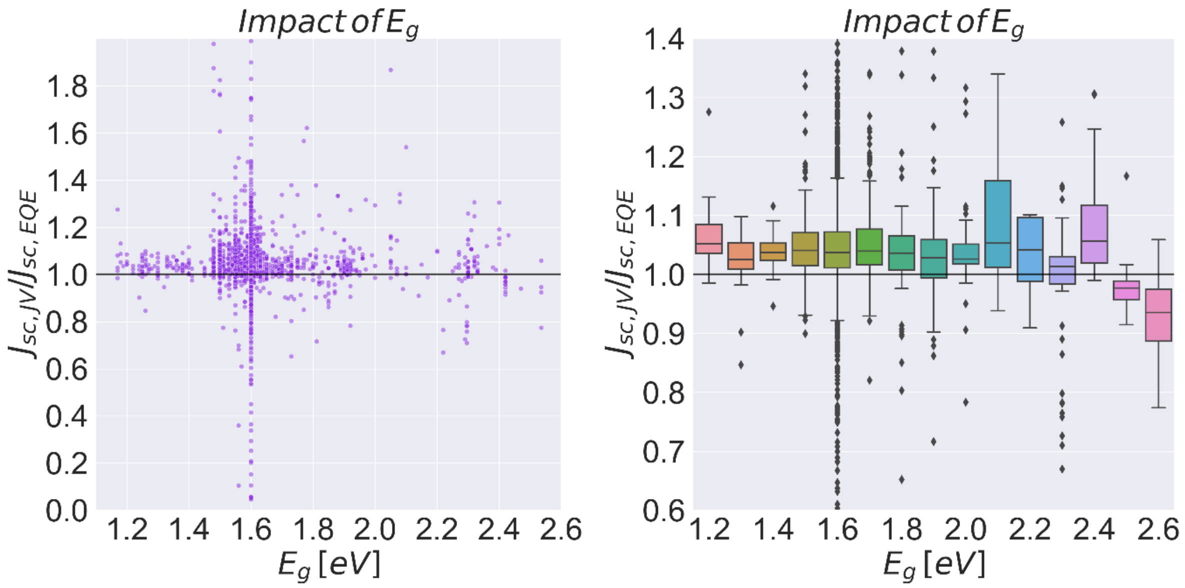

Figure S.7. Impact of perovskite band gap (Left) $J_{sc,JV}/J_{sc,EQE}$ against the perovskite band gap. (Right) Boxplot of $J_{sc,JV}/J_{sc,EQE}$ with respect to the perovskite band gap. The bin size is 0.1 eV.

In fig. S.8 a boxplot of $J_{sc,JV}/J_{sc,EQE}$ is given for the most commonly used hole transport layers. In Fig. S. 9. The corresponding plot is given for the most common electron transport layers. The same average values for $J_{sc,JV}/J_{sc,EQE}$ are observed for all common hole and electron transport layers. That is also true for both nip and pin device architectures (fig. S.10). Only for stack layers with few reported cells a larger spread in values is observed. That is statistically expected, but even there the average $J_{sc,JV}/J_{sc,EQE}$ values are positive.

In fig. S.11, the boxplot for $J_{sc,JV}/J_{sc,EQE}$ is given for 16 of the most common perovskite families, *i.e.*, a specific combination of *A*, *B*, and *C*-site ions. Approximately the same average $J_{sc,JV}/J_{sc,EQE}$ is seen for all common perovskite compositions. The compositions could be further divided based on the relative fraction between for example the different *A*-site ions when there is more than one, but the similarity in behaviour with respect to the $J_{sc,JV}/J_{sc,EQE}$ values does not merit such a subdivision.

In fig. S.12, the boxplot for $J_{sc,JV}/J_{sc,EQE}$ is given for the most common perovskite deposition procedures. This is where we have found the largest spread in the data, but also here a deviation from an average $J_{sc,JV}/J_{sc,EQE}$ of around 1.04 is only seen in cases where the data points are rather few.

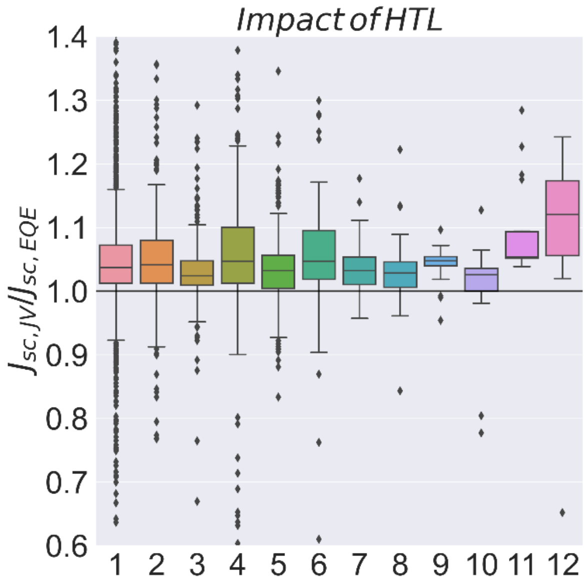

Key	HTL	Counts	Median
1	Spiro-MeOTAD	2509	1.047
2	PEDOT:PSS	720	1.024
3	PTAA	394	1.038
4	No HTL	333	1.054
5	NiO-c	307	1.032
6	P3HT	98	1.042
7	NiO-np	98	1.032
8	NiO	33	1.028
9	P3CT-Na	32	1.047
10	CuSCN	26	1.026
11	P3CT-N	24	1.048
12	CuPc	24	1.120

Figure S.8. Impact of the hole transport layer, HTL. Boxplot of $J_{sc,JV}/J_{sc,EQE}$ with respect to the most commonly used HTL.

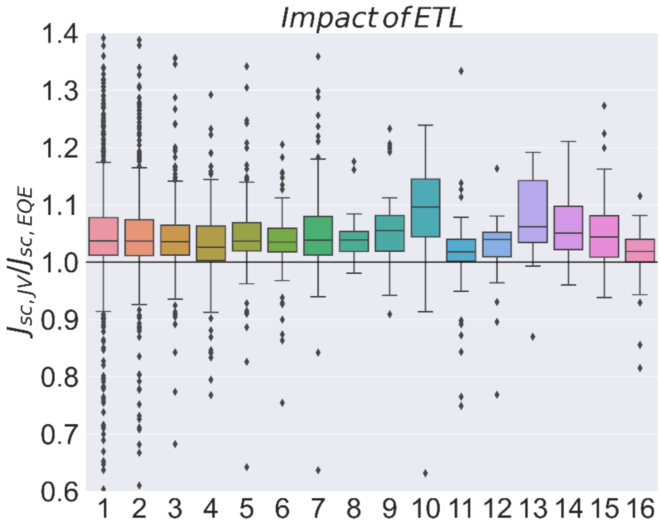

key	ETL	Counts	Median
1	TiO_2-c | TiO_2-mp	1299	1.037
2	TiO_2-c	824	1.036
3	PCBM-60 | BCP	484	1.035
4	PCBM-60	402	1.026
5	SnO_2-np	369	1.037
6	C60 | BCP	262	1.035
7	SnO2-c	206	1.039
8	PCBM-60 | C60 | BCP	60	1.039
9	PCBM-60 | ZnO-np	56	1.055
10	TiO_2-c | TiO_2-mp | ZrO_2-mp	52	1.096
11	ZnO-c	50	1.018
12	C60	49	1.039
13	PCBM-60 | Zr(acac)$_4$	40	1.062
14	TiO_2-np	38	1.051
15	TiO_2-c | PCBM-60	38	1.044
16	TiO_2-c | TiO_2-nw	36	1.019

Figure S.9. Impact of the electron transport layer, HTL. Boxplot of $J_{sc,JV}/J_{sc,EQE}$ with respect to the most commonly used ETL.

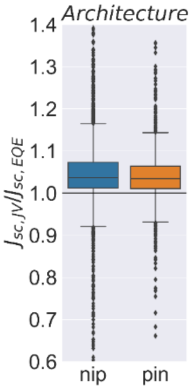

Figure S.10. Impact of the device architecture. Boxplot of $J_{sc,JV}/J_{sc,EQE}$ comparing cells with the nip (i.e. normal) and the pin (i.e. inverted) architecture.

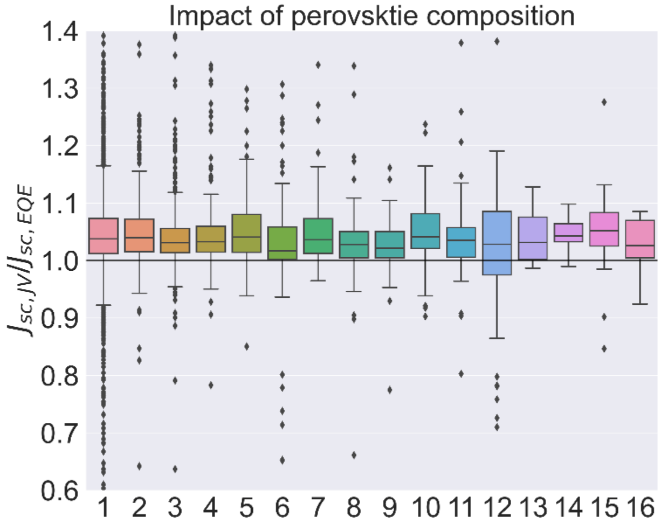

key	Perovskite composition	Counts	Median
1	MAPbI	3109	1.052
2	CsFAMAPbBrI	585	1.040
3	FAMAPbBrI	491	1.031
4	CsPbBrI	207	1.032
5	FAMAPbI	158	1.041
6	CsPbBr	97	1.017
7	FAPbI	91	1.036
8	CsFAPbBrI	71	1.028
9	CsFAPbI	60	1.021
10	MAPbBrI	56	1.041
11	CsPbI	55	1.035
12	MAPbBr	43	1.028
13	FASnI	33	1.031
14	FAMAPbSnI	32	1.043
15	MAPbSnI	25	1.038
16	BAMAPbI	22	1.026

Figure S.11. Impact of the perovskite composition. Boxplot of $J_{sc,JV}/J_{sc,EQE}$ with respect to the most commonly used families of perovskite compositions.

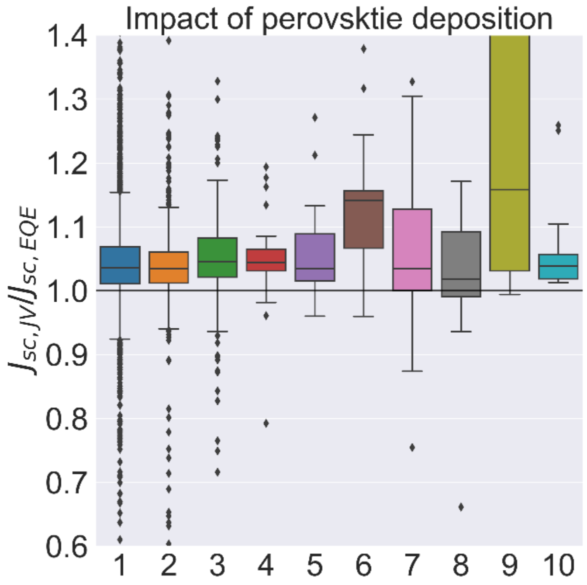

key	Deposition procedure	Counts	Median
1	Spin-coating	4019	1.036
2	Spin-coating >> Spin-coating	718	1.034
3	Spin-coating >> CBD	310	1.045
4	Doctor blading	45	1.044
5	Spin-coating >> Gas reaction	43	1.034
6	Drop-infiltration	41	1.141
7	Co-evaporation	38	1.035
8	Evaporation >> Spin-coating	23	1.018
9	Evaporation >> Gas reaction	19	1.158
10	Spin-coating >> Dipp-coating	18	1.038

Figure S.12. Impact of the perovskite deposition method. Boxplot of $J_{sc,JV}/J_{sc,EQE}$ with respect to the most commonly used perovskite deposition procedures

In fig. s.13, the stabilised efficiency PCE_{stab} is compared to the efficiency extracted from JV data, PCE_{JV}. The stabilised efficiency, ideally measured under maximum power point tracking, is a steady state measurement under operational conditions and thus considered as a better measure of the true efficiency of a cell than what a dynamic JV-scan can provide. Not surprisingly, the PCE_{stab} is lower than PCE_{JV}. In the right figure the distribution of PCE_{JV}/PCE_{stab} is given where it is seen that the median value of the quotient is 1.024. This discrepancy is in the same direction but smaller than the average discrepancy between the $J_{sc,JV}$ and the $J_{sc,EQE}$ which was found to be 1.050.

Efficiency and short circuit current is not the same thing but as there is a discrepancy between data from JV-measurements and data from both EQE and stabilised efficiency measurements it is interesting to compare the two discrepancies. In fig. S.14 the two discrepancies, i.e. PCE_{JV}/PCE_{stab} and $J_{sc,JV}/J_{sc,EQE}$ are compared. The median value of the quotient between those two quotients are 1.016 indicating that they co-vary. The EQE and the stabilised efficiency may thus be a better pair of measurements to use for checking for consistency than any of those together with JV-measurements.

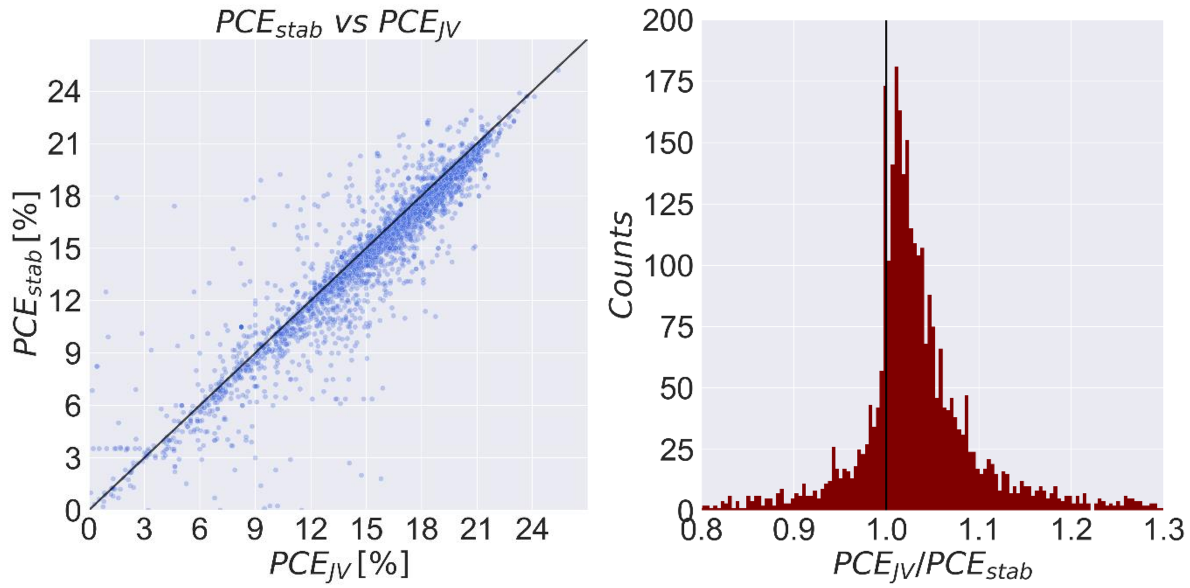

Figure S.13. (Left) Cell efficiency from JV-measurements, PCE_{JV}, against Stabilised efficiencies, PCE_{stab}, for all 3367 devices found in the Perovskite Database where both values are reported. The black diagonal line represents PCE_{JV}, = PCE_{stab}, (right) Distribution of PCE_{JV}/PCE_{stab}, for the entire dataset. The bin size is set to 0.004.

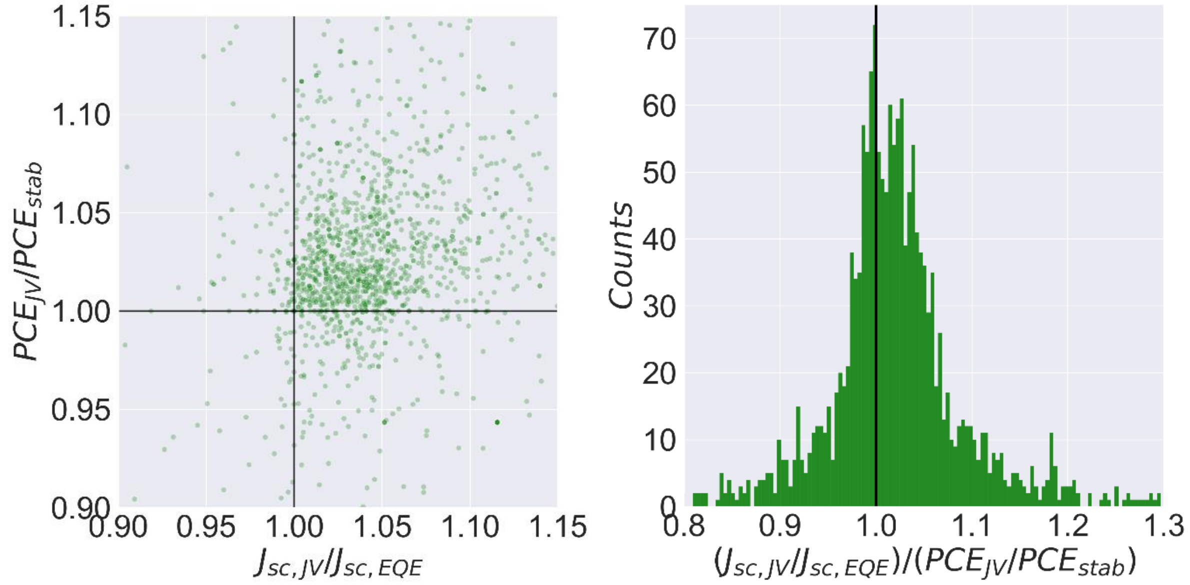

Figure S.14. (Left) PCE_{JV}/PCE_{stab} vs $J_{sc,JV}/J_{sc,EQE}$ for all 1706 datapoints that have all four values reported. in the Perovskite Database. (right) Distribution of ($J_{sc,JV}/J_{sc,EQE}$)/(PCE_{JV}/PCE_{stab}) for the entire dataset. The bin size is set to 0.004.

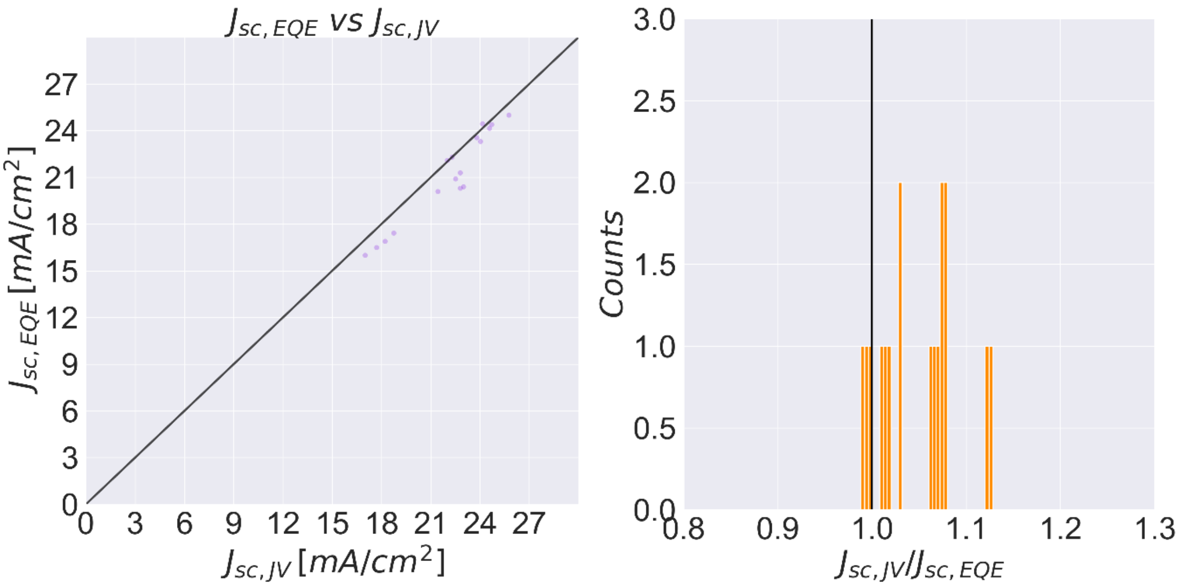

Figure S.15. A version of figure 1 in the main manuscript but which only is using externally certified data.
